# Supplementary material for: Expression perceptive fields explain individual differences in the recognition of facial emotions
Source: Commun Psychol. 2024 Jul 3;2:62. doi: 10.1038/s44271-024-00111-7 (PMC11332168; doi:10.1038/s44271-024-00111-7)
Supplement: Supplementary file 2 — Supplementary Information [file 44271_2024_111_MOESM2_ESM.pdf]

Expression perceptive fields explain individual differences in the recognition of facial emotions – Supplementary Information

Tom Murray[1,2*], Nicola Binetti[3,4], Raghav Venkataramaiyer[5], Vinay Namboodiri[5], Darren Cosker[5,6], Essi Viding[7] & Isabelle Mareschal[2]

[1]Department of Psychology, University of Cambridge, Cambridge, United Kingdom

[2]Department of Psychology, Queen Mary University of London, London, United Kingdom

[3]Department of Cognitive Neuroscience, International School for Advanced Studies, Trieste, Italy

[4]Dipartimento di Medicina dei Sistemi, Università degli studi di Roma Tor Vergata, Rome, Italy

[5]Department of Computer Science, University of Bath, Bath, United Kingdom

[6]Mixed Reality & AI Lab – Cambridge, Microsoft, Cambridge, United Kingdom

[7]Division of Psychology and Language Sciences, University College London, London, United Kingdom

Supplementary Note 1

Trace comparisons

We compared the trace of the covariances matrices (estimates for spread of perceptive fields) between each pair of emotion categories, using paired-samples Wilcoxon tests (two-sided). Normality was checked with a Shapiro-Wilk test. Results showed significant differences among all pairs of emotion categories, where perceptive fields for anger were largest, followed by fear, then sadness, then happiness.

We also ran a linear mixed effect model for each pair of emotion categories (with emotion category and the difference between the number of selected faces in each category as fixed effects, and subject as a random effect), and found that the emotion category predicted the trace, even after controlling for any differences in the number of selected faces. Results of these tests are reported in Supplementary Table 1.

Supplementary Table 1: Trace (estimates of spread) of perceptive fields for each emotion category

Spread estimates	
Angry	Mean = 3.782, S.D. = 1.682; Shapiro-W = 0.936, $p < .001$
Fear	Mean = 3.109, S.D. = 1.371; Shapiro-W = 0.923, $p < .001$
Happy	Mean = 2.592, S.D. = 1.105; Shapiro-W = 0.930, $p < .001$
Sad	Mean = 2.846, S.D. = 1.153; Shapiro-W = 0.930, $p < .001$
Pairwise comparisons	
Angry-Fear	$W(291) = 13473$, $p < .001$, $r_{rb} = .370$, 95% CI [0.223, 0.459]
	Mixed-LM expression coefficient = 0.673, $p < .001$
Angry-Happy	$W(291) = 7486$, $p < .001$, $r_{rb} = .650$, 95% CI [0.514, 0.697]
	Mixed-LM expression coefficient = 1.191, $p < .001$
Angry-Sad	$W(291) = 9268$, $p < .001$, $r_{rb} = .567$, 95% CI [0.361, 0.586]
	Mixed-LM expression coefficient = 0.936, $p < .001$
Fear-Happy	$W(291) = 13887$, $p < .001$, $r_{rb} = .351$, 95% CI [0.197, 0.438]
	Mixed-LM expression coefficient = 0.518, $p < .001$
Fear-Sad	$W(291) = 17377$, $p = .005$, $r_{rb} = .188$, 95% CI [0.031, 0.286]
	Mixed-LM expression coefficient = 0.263, $p = .003$
Happy-Sad	$W(291) = 17132$, $p < .001$, $r_{rb} = .199$, 95% CI [0.044, 0.313]
	Mixed-LM expression coefficient = 0.254, $p = .001$

Supplementary Note 2

Trace correlations

Perceptive field sizes were correlated with each other, suggesting that some individuals have larger perceptive fields across the different emotion categories. Supplementary Table 2 presents the results of the Spearman's correlations (two-sided).

Supplementary Table 2: Spearman's correlations between the spreads of the perceptive fields

Emotion pair	*Correlation*
Angry-Fear	rho(290)=0.177, p=.002, 95%CI [-0.123, 0.107]
Angry-Happy	rho(290)=0.146, p=.013, 95%CI [-0.115, 0.112]
Angry-Sad	rho(290)=0.263, p<.001, 95%CI [-0.109, 0.113]
Fear-Happy	rho(290)=0.211, p<.001, 95%CI [-0.110, 0.114]
Fear-Sad	rho(290)=0.252, p<.001, 95%CI [-0.109, 0.113]
Happy-Sad	rho(290)=0.212, p<.001, 95%CI [-0.117, 0.118]

Supplementary Note 3

Weighting KDE by generation

The participant's responses to the expressions were binary (i.e. faces were selected/not-selected), and we did not ask participants for subjective ratings of each expression across iterations, we only obtained this on the last iteration. One might consider, however, that more accurate models of perceptive fields might be gained by weighting the contribution of the expressions to the KDE by the fit of the expression to the participant's representation.

One such method would be to weight the KDE by the generation number, as we showed in Binetti et al (2022, PNAS) that expressions converge to the participant's preferred expression as generations increase. We therefore ran the same prediction analyses again, weighting the contribution of each expression to each KDE by the generation that the expression appeared in, and the pattern of results were near-identical.

Predictive accuracy:

All emotions:	Mean = 0.454, S.D. = 0.096; t(33)=12.201, p<.001, d=2.124, 95% CI [1.660, 3.060]
Angry:	Mean = 0.454, S.D. = 0.317; t(33)=3.707, p<.001, d=0.645, 95% CI [0.329, 1.050]
Fear:	Mean = 0.326, S.D. = 0.275; t(33)=1.584, p=.061, d=0.276, 95% CI [-0.057, 0.622]
Happy:	Mean = 0.575, S.D. = 0.307; t(33)=6.072, p<.001, d=1.057, 95% CI [0.697, 1.550]
Sad:	Mean = 0.408, S.D. = 0.276; t(33)=3.291, p=.001, d=0.573, 95% CI [0.249, 0.951]

Within vs between subject predictions:
Within-subject mean = 45.4%
Between-subject mean = 43.4%
Probability between > within: p=.001
Probability between < chance: p<.001

Overlap/agreement correlation:
Rho=.223, p(perm)<.001, 95% CI [-0.086, 0.079]

The main difference between these set of results and the results reported in the main text is that the prediction of the fear label is no longer significant. However, treating the data points as binary when modelling the KDE better reflects the selection process, and makes no assumptions about the fit of each face to the participant's representation.

Supplementary Note 4

Predictions of ‘average perceptive fields’

Creating new probability density functions as averages of individual functions (in different spaces, with different shapes), poses a difficult challenge. Below we report the results of several attempts at generating predictions of an average KDE.

As the area under the KDE always sums to 1, one approach could be to fit a KDE for each emotion category, over a whole dataset concatenated across the data from individuals. However in testing, the categorisations predicted by these averaged KDEs were no higher than chance:

> True accuracy: 45.9%, S.D. = 9.7
> Accuracy (averaged KDE): Mean = 25.4%, S.D. = 3.4
> True vs average: $t(33)=10.369$, $p<.001$, $d= 0.801$, 95% CI [0.002, 0.179]
> Average vs chance: $t(33)=0.616$, $p=0.271$, $d= 0.107$, 95% CI [-0.204, 0.53]

As the KDE was fit across multiple individual datasets (that lie in different locations, and have different sizes), it is not unexpected that this attempt fails. We next tried the same approach, but scaled each individual dataset so that the mean and variance of each set of principal components for each emotion category were equal to the average mean and variance across individuals. Essentially, this approach moved all perceptive fields so that the mean was placed at the average expression chosen across all individuals, and adjusted the variance so that the size was approximately equal across individuals. Again, this approach performed no better than chance:

> True accuracy: 45.9%, S.D. = 9.7
> Accuracy (averaged KDE): Mean = 25.4, S.D. = 3.7
> True vs average: $t(33)=10.108$, $p<.001$, $d=0.767$, 95% CI [0.002, 0.166]
> Average vs chance: $t(33)=1.487$, $p=0.073$, $d=0.259$, 95% CI [-0.115, 0.705]

Finally, we tried an approach where predictions of an ‘average’ were generated by taking the average density across all individual KDEs for each test expression, and using these averaged densities to predict the labels:

> True accuracy = 45.9%, S.D. = 9.7
> Accuracy (averaged KDE): Mean = 26.2%, S.D. = 2.9
> True vs average: $t(33)=10.414$, $p<.001$, $d= 0.782$, 95% CI [0.002, 0.178]
> Average vs chance: $t(33)=2.368$, $p=.012$, $d= 0.412$, 95% CI [0.085, 0.809]

Although this approach did not explicitly define an average KDE, the averaged predictions are above chance (but weak), suggesting this approach is valid at generating predictions of an ‘average perceptive field’. Additionally, the true (within-subject) predictive accuracy was larger than this averaged predictive accuracy, supporting our claim that perceptive fields are unique to the individual.
